# Acute Cardiac Tamponade as a Complication of Pulmonary Vein Isolation Ablation

**DOI:** 10.7759/cureus.19572

**Published:** 2021-11-14

**Authors:** Jeffrey Tsai, Nathaniel Chishinga, Shibinath Velutha Mannil, Robin Schaffer, Andrzej Kuchciak, Sabas I Gomez, John Dylewski, John Sciarra

**Affiliations:** 1 Anesthesiology, Larkin Community Hospital, South Miami, USA; 2 Internal Medicine, Larkin Community Hospital Palm Springs Campus, Hialeah, USA; 3 Internal Medicine, Larkin Community Hospital, South Miami, USA; 4 Cardiology, Larkin Community Hospital, South Miami, USA

**Keywords:** cardiac tamponade, radiofrequency ablation, beck's triad, pulmonary vein, atrial fibrillation recurrence, pericardial effusion

## Abstract

Perioperative acute cardiac tamponade associated with perforation from pulmonary vein isolation (PVI) and radiofrequency catheter ablation (RFCA) for the treatment of refractory atrial fibrillation (AF) is rare. If not identified early and managed promptly, it can lead to decreased ejection fraction, hypotension, and ultimately death. We report a case of acute tamponade that was diagnosed and successfully managed following PVI and RFCA. A 49-year-old woman with a past medical history of paroxysmal AF and sick sinus syndrome presented to our hospital with intermittent episodes of palpitations and recurrent episodes of syncope. Given the drug-refractory AF, our patient underwent PVI and RFCA. A loop recorder was implanted for recurrent episodes of syncope, which revealed that she had sick sinus syndrome. During the current visit, transthoracic ECG revealed mild tricuspid regurgitation and trace pericardial effusion. Her left ventricle (LV) ejection fraction was 60%. A CT angiography of the pulmonary vessels and the aorta showed no evidence of pulmonary embolism, aortic aneurysm, or aortic dissection. However, there was an enlarged heart size and small bilateral pleural effusions. During a second PVI and RFCA, while in the operating room, the patient became hypotensive. A transesophageal echocardiogram (TEE) showed diastolic volume reduction in the right atrium and right ventricular and pericardial effusion. Intravenous (IV) resuscitation with lactated Ringer's solution and saline solution was rapidly given to the patient while performing percutaneous pericardiocentesis. In addition, packed red blood cells were transfused into the patient, and phenylephrine was given IV. There was 400 mL of blood drained from the pericardial sac, confirming the presence of acute cardiac tamponade. Following the pericardiocentesis, the patient became normotensive. A drainage tube was inserted into the pericardial space, which drained a total of 250 mL of sanguineous fluid over the next 48 hours after the procedure, after which it was removed without signs of persistent bleeding, and the patient was discharged. We conclude that her previous PVI and RFCA, and the anatomical distortion that might have resulted from her enlarged heart size, may have predisposed her to perforation and thus acute cardiac tamponade in this PVI and RFCA. Although perforation leading cardiac tamponade is rare during PVI and RFCA, the future focus when performing this procedure should be to (i) have a high index of suspicion for acute cardiac tamponade, (ii) use TEE and intracardiac echocardiography for early detection, and (iii) promptly manage the acute cardiac tamponade with pericardiocentesis, while giving IV fluid resuscitation and positive inotropes to hemodynamically stabilize the patient.

## Introduction

Intracardiac electrophysiological studies (EPS) with or without radiofrequency catheter ablation (RFCA) are an essential component in the diagnosis and therapeutic workup of drug-refractory arrhythmias [1]. EPS involves the percutaneous introduction of one or more catheters to record the heart's electrical activity and identify the source of the arrhythmias. This includes isolating the four pulmonary veins and identifying the triggers for atrial fibrillation (AF). Pulmonary vein isolation (PVI) and RFCA following EPS is a class IA recommendation for treating refractory paroxysmal and symptomatic AF that is not responding to at least one class I or III antiarrhythmic medication [1,2]. RFCA aims to eliminate triggers and reentry circuits that initiate, perpetuate, and sustain AF [3]. In a systematic review, the incidence of procedure-related deaths was reported to be 0.06%, atrioesophageal fistula 0.1%, and phrenic nerve injury 0.4% [3]. PVI and RFCA can, however, be challenging, requiring considerable procedural time and operator expertise, and is limited by whether or not the provocation of the pulmonary vein ectopy that is causing the AF can be achieved and reproduced during the procedure [4]. Major complications that are life-threatening or cause permanent harm and require intervention or prolonged hospitalization can occur among those undergoing RFCA [5]. These complications include pulmonary vein stenosis, stroke/transient ischemic attack, pulmonary embolism, cardiac perforation and tamponade, pericardial effusion, arterial injury, thrombophlebitis, and systemic arterial embolism [5,6].

Acute cardiac tamponade following PVI and RFCA is relatively uncommon with an incident rate between 0.6% and 1.3% [5,7] but decreases with experienced operators [8-10]. Despite its low incidence, it is a serious complication that requires immediate percutaneous pericardiocentesis to prevent sudden cardiac death. Therefore, prompt diagnosis and management of acute cardiac tamponade can result in a good outcome [11]. We report the case of a woman with refractory AF who developed acute cardiac tamponade during PVI and RFCA and how this was managed.

## Case presentation

A 49-year-old woman with a past medical history of hypertension, transient ischemic attack, paroxysmal AF, and sick sinus syndrome presented to our hospital with intermittent episodes of palpitations and recurrent episodes of syncope. She had initially presented to our hospital seven months ago with intermittent episodes of palpitations for three months, with documented electrocardiographic (ECG) evidence of AF. She had previously been on oral 5 mg pindolol but continued to experience paroxysmal AF. Given the drug-refractory AF, our patient at that time underwent PVI and RFCA from which she was discharged 24 hours after the procedure. She was again seen at our hospital three months ago for evaluation of recurrent episodes of syncope, of which a loop recorder was implanted, which revealed that she had sick sinus syndrome. Her essential hypertension was diagnosed approximately 10 years ago, for which she was taking oral 10 mg lisinopril once daily and 5 mg pindolol once daily. Her transient ischemic attack was diagnosed 12 months ago, for which she was taking apixaban 2.5 mg twice daily. She had a parathyroidectomy approximately 10 years ago, and a repair of a patent foramen ovale 10 months ago.

On examination, she was hemodynamically stable. Examinations of the neck, heart, and lung and auscultations were unremarkable. Transthoracic ECG revealed mild tricuspid regurgitation and trace pericardial effusion. There was no evidence of left ventricular (LV) hypertrophy. Her LV ejection fraction (EF) was 60% and there were no regional wall motion abnormalities of her LV. There was no evidence of thrombus in any of the cardiac chambers. A CT angiography of the pulmonary vessels and the aorta showed no evidence of pulmonary embolism, aortic aneurysm, or aortic dissection. However, there was an enlarged heart size and small bilateral pleural effusions.

**Figure 1:**
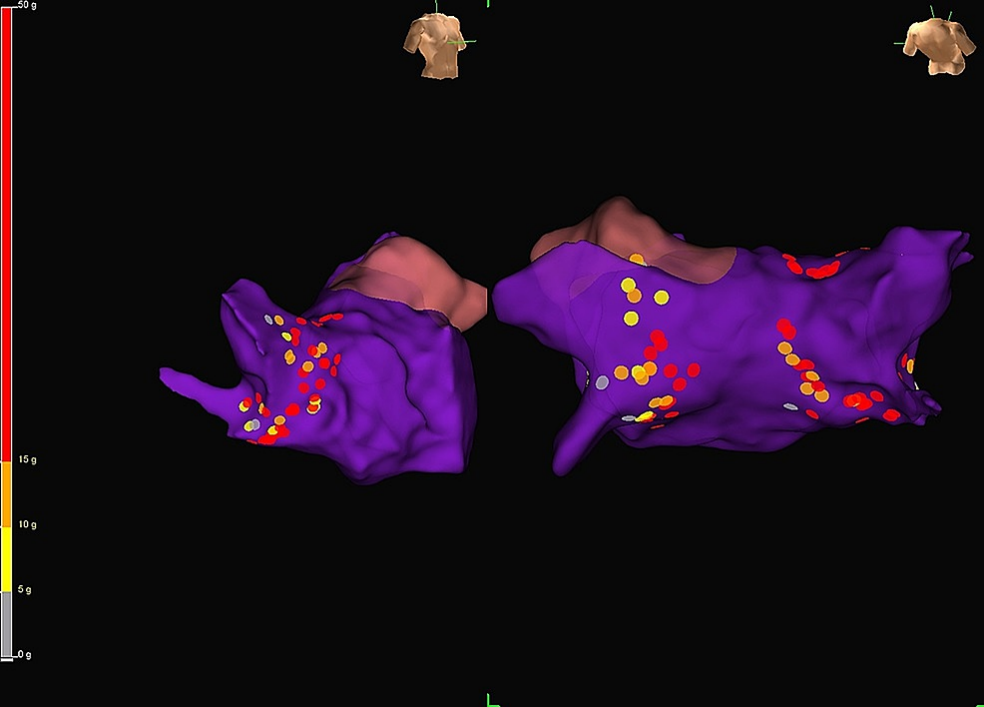
Three-dimensional reconstruction of the left atrium of the patient after isolating the pulmonary veins by encircling the left and right pulmonary veins with the ablated lesions. The left figure shows the anterior-lateral view of the right superior and inferior pulmonary veins. The right figure shows the posterior-anterior view. The dots indicate the sites of radiofrequency catheter ablation.

Given the sequence of events and the refractory AF, second PVI and RFCA were scheduled for our patient. In the operating room (OR), the patient was prepared with an arterial line in her right radial artery, two 18-gauge intravenous (IV) cannulas were placed on her left hand, and one 20-gauge IV cannula was placed on her right hand. Intubation was achieved without difficulty. Pre-procedure transesophageal ECG (TEE) in the OR was performed and was unremarkable with no significant pericardial effusion or tamponade seen. The patient underwent induction using rocuronium, propofol, and sevoflurane and was then maintained on a combined general anesthetic consisting of propofol infusion and sevoflurane nitrous oxide. She remained hemodynamically stable throughout the PVI and RFCA, requiring ephedrine 10 mg IV for blood pressure support after induction of general anesthesia. The three-dimensional EnSite Precision cardiac mapping system (St. Jude Medical, Inc., Saint Paul, MN) was used to isolate the pulmonary veins during EPS and to perform circumferential point-by-point linear RFCA (Figure 1).

**Figure 2:**
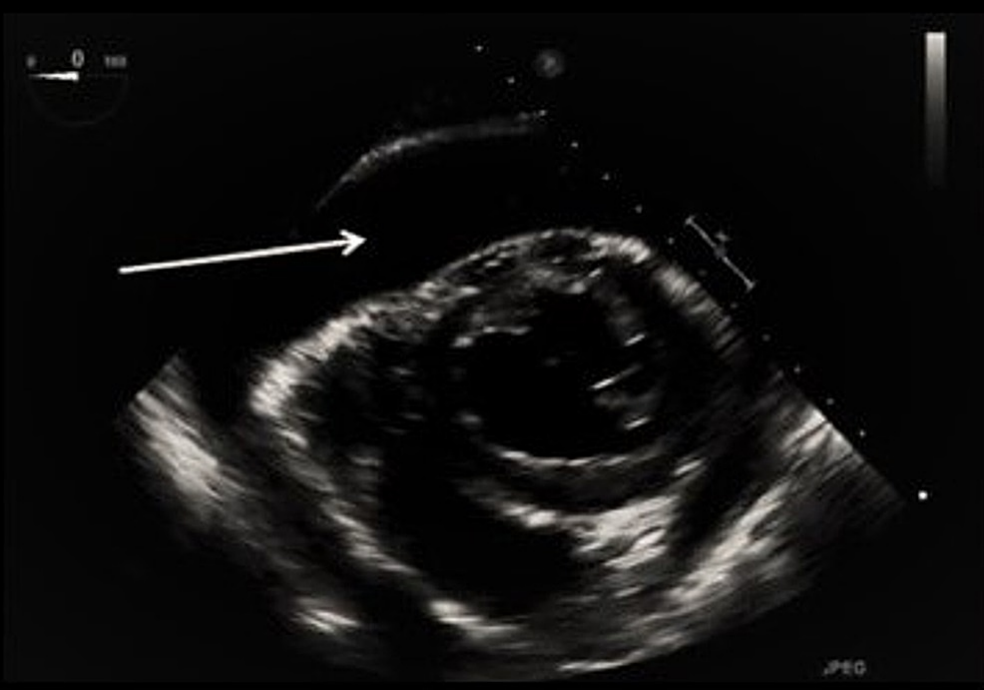
Bedside transesophageal echocardiogram (TEE) with pericardial effusion A transgastric short-axis view demonstrated right atrial and right ventricular collapse during the majority of the cardiac cycle with a significant reduction of venous flow.

After the PVI and RFCA were completed and verified, the patient became hypotensive. The bedside TEE showed diastolic volume reduction in the right atrium and right ventricular, and pericardial effusion (Figure 2). IV resuscitation with 1000 mL lactated Ringer's solution and 1000 mL 0.9% saline solution was rapidly given to the patient while performing percutaneous pericardiocentesis. In addition, two units of packed red blood cells were transfused into the patient, and a total of 0.5 mg phenylephrine was given IV. There was 400 mL of blood drained from the pericardial sac, confirming the presence of acute cardiac tamponade. Following the pericardiocentesis, the patient became normotensive. A drainage tube was inserted into the pericardial space, which drained a total of 250 mL of sanguineous fluid over the next 48 hours after the procedure; after which, it was removed without signs of persistent bleeding, and the patient was discharged.

## Discussion

Our patient presented with refractory paroxysmal AF and had to undergo a second PVI and RFCA, despite a previous attempt. This was not unexpected because, unlike supraventricular tachycardias, patients with paroxysmal AF usually have multiple pulmonary vein foci that trigger the AF and in more than one pulmonary vein. Many of these foci originate distally in the pulmonary veins. Also, direct catheter ablation of these foci can be limited by (i) paucity of spontaneous or inducible arrhythmias during the procedure and (ii) the infrequency, with which AF initiation can be reproducibly triggered during the procedure [11]. Furthermore, around 3% of paroxysmal AF has no inducible ectopy-initiating AF during EPS [12]. Thus, abolishing these initiating triggers may be challenging, requiring that the procedure is repeated to successfully treat the paroxysmal AF, as was the case in our patient.

Acute cardiac tamponade following PVI and RFCA is relatively uncommon, especially with experienced operators. The previous PVI and RFCA in our female patient could have predisposed her to perforation during this second procedure due to weakness and scar tissue that might have formed in the pulmonary veins. Also, an enlarged heart size on CT angiogram with mild tricuspid regurgitation on TEE suggests that our patient had a dilated heart that might have distorted our patient’s heart anatomy, thus confounding the RFCA and increasing the risk of perforation and cardiac tamponade [13].

One study showed a clear association between the onset of acute cardiac tamponade and transseptal puncture or mechanical injury to the left atrium in addition to a high level of anticoagulation [14]. The source of cardiac tamponade in our patient could have been perforation of the proximal ends of the pulmonary veins of the left atrium. This led to rapid accumulation of blood into the oblique pericardial sinus causing cardiac tamponade and increased intrapericardial pressure that compressed the cardiac chambers, especially the right atrium and right ventricle. Compression of the right atrium and right ventricle resulted in decreased venous return. More still, compression led to an increase in right ventricular pressure that pushed the septum to the left ventricle causing a decrease in ejection fraction and hypotension.

Our patient became hypotensive following RFCA and did not show the classic Beck’s triad of muffled heart sounds, hypotension, and jugular venous distension for cardiac tamponade [15]. A TEE was expeditiously performed on our patient that identified a pericardial effusion and emergency pericardiocentesis was performed which confirmed the cardiac tamponade (Figure 2). The classic signs in Beck’s triad are infrequently present in acute cardiac tamponade, which underscores the importance of using TEE and intracardiac echocardiography during PVI and RFCA for early detection and management of acute cardiac tamponade [16]. The degree and type of perforation following RFCA determine whether or not a surgical repair is required [17]. A surgeon was on standby in the event that the TEE showed perforation that required repair, but this did not happen in our patient. In the majority of patients that develop acute cardiac tamponade following RFCA, emergency pericardiocentesis is sufficient for complete recovery, but an urgent surgical repair may be lifesaving in 15-20% of the cases; raising the question of the safety of the procedure performed without in-house surgical backup [18,19]. In addition to emergency pericardiocentesis, blood was transfused, positive inotropes, and IV fluids were given to hemodynamically stabilize our patient. Vigorous hydration with lactated Ringer's solution and 0.9% saline solution was given to our patient to maintain right ventricular filling pressure and thus prevent right ventricular collapse in the setting of cardiac tamponade.

## Conclusions

Although rare, acute cardiac tamponade may occur in patients undergoing PVI and RFCA. The degree and type of perforation following RFCA determine whether or not a surgical repair is required. Previous PVI and RFCA and anatomical distortion of the heart may further increase the risk of perforation and thus acute cardiac tamponade in subsequent procedures. The classic Beck’s triad signs for cardiac tamponade may not be readily present in acute cardiac tamponade arising from PVI and RFCA, as such, a high index of suspicion for acute cardiac tamponade is required in a patient that becomes hemodynamically unstable during the procedure. Prompt diagnosis using TEE and intracardiac echocardiography, and emergency pericardiocentesis can improve the outcome in the patient. In addition to emergency pericardiocentesis, blood transfusion, IV fluids, and positive inotropes may be required to stabilize the patient hemodynamically.

## References

[1] Calkins H, Kuck KH, Cappato R (2012). 2012 HRS/EHRA/ECAS expert consensus statement on catheter and surgical ablation of atrial fibrillation: recommendations for patient selection, procedural techniques, patient management and follow-up, definitions, endpoints, and research trial design. J Interv Card Electrophysiol.

[2] Blanc JJ, Almendral J, Brignole M (2008). Consensus document on antithrombotic therapy in the setting of electrophysiological procedures. Europace.

[3] Gupta A, Perera T, Ganesan A (2013). Complications of catheter ablation of atrial fibrillation: a systematic review. Circ Arrhythm Electrophysiol.

[4] Ziad I, Miller J, Zipes D (2012). Atrial fibrillation. Clinical Arrhythmology and Electrophysiology: A Companion to Braunwald's Heart Disease.

[5] Issa Z, Miller J, Zipes D (2018). Clinical Arrhythmology and Electrophysiology. J Cardiovasc Electrophysiol.

[6] Dagres N, Hindricks G, Kottkamp H (2009). Complications of atrial fibrillation ablation in a high-volume center in 1,000 procedures: still cause for concern?. J Cardiovasc Electrophysiol.

[7] Horowitz LN, Kay HR, Kutalek SP, Discigil KF, Webb CR, Greenspan AM, Spielman SR (1987). Risks and complications of clinical cardiac electrophysiologic studies: a prospective analysis of 1,000 consecutive patients. J Am Coll Cardiol.

[8] Bertaglia E, Zoppo F, Tondo C (2007). Early complications of pulmonary vein catheter ablation for atrial fibrillation: a multicenter prospective registry on procedural safety. Heart Rhythm.

[9] Russo AM, Zeitler EP, Giczewska A (2021). Association between sex and treatment outcomes of atrial fibrillation ablation versus drug therapy: results from the CABANA trial. Circulation.

[10] Michowitz Y, Rahkovich M, Oral H (2014). Effects of sex on the incidence of cardiac tamponade after catheter ablation of atrial fibrillation: results from a worldwide survey in 34,943 atrial fibrillation ablation procedures. Circ Arrhythm Electrophysiol.

[11] O'Connor J, Ditillo M, Scalea T (2009). Penetrating cardiac injury. J R Army Med Corps.

[12] Pak HN (2019). Catheter ablation of long-standing persistent atrial fibrillation: a reckless challenge or a way to real cure?. Korean Circ J.

[13] Khan MN, Jaïs P, Cummings J (2008). Pulmonary-vein isolation for atrial fibrillation in patients with heart failure. N Engl J Med.

[14] Lan L, Zeng Y, Wang WR (2013). Clinical characteristics and risk factors of pericardial effusion complicating radiofrequency catheter ablation in Chinese Han patients with tachyarrhythmias. Herz.

[15] Haegeli LM, Wolber T, Ercin E (2010). Double transseptal puncture for catheter ablation of atrial fibrillation: safety of the technique and its use in the outpatient setting. Cardiol Res Pract.

[16] Cappato R, Calkins H, Chen SA (2009). Prevalence and causes of fatal outcome in catheter ablation of atrial fibrillation. J Am Coll Cardiol.

[17] Hamaya R, Miyazaki S, Taniguchi H (2018). Management of cardiac tamponade in catheter ablation of atrial fibrillation: single-centre 15 year experience on 5222 procedures. Europace.

[18] Tao T, Zheng J, Xu H, Ni Y (2019). An abnormal left ventricular-atrial perforation after radiofrequency catheter ablation: a case report. J Cardiothorac Surg.

[19] Mujović N, Marinković M, Marković N (2016). Management and outcome of periprocedural cardiac perforation and tamponade with radiofrequency catheter ablation of cardiac arrhythmias: a single medium-volume center experience. Adv Ther.

